# Transductive learning as an alternative to translation initiation site identification

**DOI:** 10.1186/s12859-017-1502-6

**Published:** 2017-02-02

**Authors:** Cristiano Lacerda Nunes Pinto, Cristiane Neri Nobre, Luis Enrique Zárate

**Affiliations:** 1 School of Engeneering of Minas Gerais - EMGE, Belo Horizonte, 30150-250 Brazil; 2 Pontifical Catholic University of Minas Gerais - PUC-MG, 255, Walter Ianni Street, Belo Horizonte, 31980-110 Brazil

**Keywords:** Machine learning, Transductive learning, SVM, TSVM, Translation initiation site, mRNA

## Abstract

**Background:**

The correct protein coding region identification is an important and latent problem in the molecular biology field. This problem becomes a challenge due to the lack of deep knowledge about the biological systems and unfamiliarity of conservative characteristics in the messenger RNA (mRNA). Therefore, it is fundamental to research for computational methods aiming to help the patterns discovery for identification of the Translation Initiation Sites (TIS). In the field of Bioinformatics, machine learning methods have been widely applied based on the inductive inference, as Inductive Support Vector Machine (ISVM). On the other hand, not so much attention has been given to transductive inference-based machine learning methods such as Transductive Support Vector Machine (TSVM). The transductive inference performs well for problems in which the amount of unlabeled sequences is considerably greater than the labeled ones. Similarly, the problem of predicting the TIS may take advantage of transductive methods due to the fact that the amount of new sequences grows rapidly with the progress of Genome Project that allows the study of new organisms. Consequently, this work aims to investigate the transductive learning towards TIS identification and compare the results with those obtained in inductive method.

**Results:**

The transductive inference presents better results both in *F-measure* and in *sensitivity* in comparison with the inductive method for predicting the TIS. Additionally, it presents the least failure rate for identifying the TIS, presenting a smaller number of False Negatives (FN) than the ISVM. The ISVM and TSVM methods were validated with the molecules from the most representative organisms contained in the RefSeq database: *Rattus norvegicus*, *Mus musculus*, *Homo sapiens*, *Drosophila melanogaster* and *Arabidopsis thaliana*. The transductive method presented *F-measure* and *sensitivity* higher than 90% and also higher than the results obtained with ISVM. The ISVM and TSVM approaches were implemented in the TransduTIS tool, TransduTIS-I and TransduTIS-T respectively, available in a web interface. These approaches were compared with the TISHunter, TIS Miner, NetStart tools, presenting satisfactory results.

**Conclusions:**

In relation to precision, the results are similar for the ISVM and TSVM classifiers. However, the results show that the application of TSVM approach ensured an improvement, specially for *F-measure* and *sensitivity*. Moreover, it was possible to identify a potential for the application of TSVM, which is for organisms in the initial study phase with few identified sequences in the databases.

**Electronic supplementary material:**

The online version of this article (doi:10.1186/s12859-017-1502-6) contains supplementary material, which is available to authorized users.

## Background

Translation and transcription processes are used by the cells in order to interpret and express their genetic information [1]. Only a portion from the whole transcript messenger RNA (mRNA) gets translated into protein, which is called Coding Sequence (CDS). The correct protein coding region identification is one of the main problems in the molecular biology, since it motivates the search for conservative features in the mRNA sequence that enables the detection of a CDS region.

In eukaryotes, the CDS region is delimited by indicators denominated *start codon* and *stop codon*. The *start codon*, preferably identified by AUG triplet, also known as Translation Initiation Site (TIS), determines the start of the process of protein synthesis, which is one of the most important processes in the regulation of gene expression [2]. The translation process often begins in the first occurrence of an AUG codon [3], but can also begins in different codons as indicated in [4]. Similarly, the *stop codon*, identified by the occurrence of triplets UAA, UAG or UGA, determines the end of protein translation process.

The translation initiation site directly influences the produced protein, it may alter its structure and function in the cellular environment. The lack knowledge of conservative characteristics to identify the translation initiation site turns the TIS prediction into a complex problem.

**Fig. 1:**
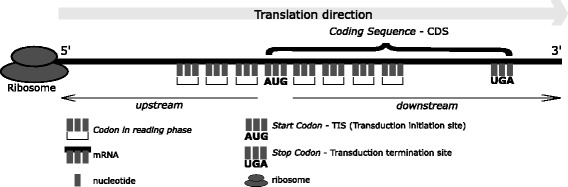
Representation of a mRNA sequence according to the scanning model in the eukaryotes

The scanning model in eukaryotes [5] assumes that the link between the ribosome and the mRNA sequence initially occurs at the 5’ and goes toward the 3’ region. In [3], the authors establish the following concepts: *upstream* and *downstream* regions and the reading phase of the mRNA sequence by the ribosome during protein production process. This process can be seen in Fig. 1.

The identification of the TIS is a non-trivial task due to the fact that the mRNA molecules possess, depending on the organism, thousands of nucleotides and that the translation process is motivated by an intracellular context of difficult simulation. Additionally, the identification process corresponds to a combinatorial computational problem in the order of 4^*n*^, where *n* is the number of nucleotides considered in the analysis.

The task of predicting the TIS can be modeled as a binary classification problem, i.e., positive sequence when a TIS is identified and negative sequence otherwise. However, the TIS prediction context induces a natural unbalance in the databases, once in each mRNA sequence there is only one AUG codon identified as *start codon* (TIS), while all other AUG codons are identified as non-TIS (nTIS). For instance, the unbalance for the organisms *Mus musculus* and *Rattus norvegicus* are 1:23 and 1:131, respectively [6]. Such unbalance can be solved by two approaches: *oversampling* and *undersampling*. *Oversampling* artificially generates samples of the minority class in order to balance the database. For instance, the SMOTE algorithm [7] makes usage of this approach, applied in order to generate positive sequences (TIS) of the minority class.

Furthermore, *undersampling* selects samples within the majority class in order to obtain approximately the amount of samples contained in the minority class. In [6], the authors introduced an *undersampling* method called *M-Clus*, which performs clustering of the samples contained in the majority class and selects the centroid or most significant elements from each cluster to integrate the database used to build the classifier. Thus, the number of clusters to be considered corresponds to the number of samples available in the minority class.

Both *oversampling* and *undersampling* approaches present problems due to the biological context modification. The first method generates artificial samples from the minority class, enabling the creation of samples possibly inconsistent with the class. Similarly, the second approach fails to consider samples from the majority class that may be relevant for classification. In order to deal with the loss of relevant information caused by *undersampling*, [6] propose a method of knowledge inclusion called *inAKnow*. This method classifies sequences from the *downstream* region using a previous model generated from sequences belonging to the *upstream* region. These new sequences are included in the final model building.

The approach used in this study avoids the unbalance problem, inherent in the TIS prediction, by not considering all the occurrences of the AUG triplet, that are not TIS, as nTIS (negative class). From the biological point of view, AUG triplets found in the same reading phase of a TIS present more similarity with this class than with the nTIS class. Such similarity was verified in [8] by studying the translation mechanism of HIV into mRNA molecules and the identification of the restarting of the translation process, occasioned by the presence of a AUG triplet near by a stop codon triplet. Under this assumption, we will use as nTIS only *upstream* AUG codon and out of reading phase with the TIS.

On the other hand, due to the good performance of the inductive SVM classifier for classification problems in different domains with high dimensionality [9], this classifier has often been used in the TIS prediction. In the experiments carried out by [10] and [11], the use of inductive SVM aiming the TIS prediction presented an accuracy gain with the use of kernel functions such as locality-improved kernel and Salzberg kernel, reaching an accuracy of 88.6% for the database used in [12]. The TIS Hunter^1^ program [13] proposes the usage of Edit Kernels function and a methodology for redundancy control in the genetic code that consists in converting the set of nucleotides from a *downstream* region into a amino acids sequence prior to the SVM training. This methodology reached 99.9% accuracy for the same database proposed in [12] and 96.7% accuracy for human mRNA from NCBI Reference Sequence (RefSeq) database [14]. Although the TISHunter predictor has presented very satisfactory results, it needs a specific kernel function. The proposed approach in this work uses the RBF function, which is a standard function in classification problems.

In addition, this tool is a TIS predictor and does not work as a classifier. In the other words, for each mRNA molecule, there is only one indication of TIS, without classification of the other AUGs of the molecule. In mistake situations, there is no indication of other possible AUGs that could be TIS. This information will be important for anyone who wants to promptly identify the beginning of translation. Besides that, in [15] the authors mention that the success of TISHunter depends on the existence of related proteins or cDNA sequences in the database. They also highlight that the Kernel function, once determined for the training set, can not be easily adapted. Therefore, there is a need for new approaches to TIS identification.

With the progress of the Genome Project [16], a greater number of molecules are sequenced and made available in the RefSeq database daily [14]. However, a small number of molecules, such as the *Nasonia vitripennis* organism, which has only 35 REVIEWED molecules available, on 22^*nd*^ April/2014, is a challenges for classification problems. In such case, the inductive inference does not posses enough information for training the model. To overcome this problem, the transductive inference, introduced by [17], represents an alternative way. The core idea behind the transductive inference is to build a classifier using two data sets: 1) the original training set, which contains the already classified data, and 2) the prediction set, in which the elements are not labeled yet. Thus, the transductive inference have more available information for training than through inductive inference, and can be considered as an alternative for solving the problem TIS prediction, in a single process step.

The transductive inference can be classified as semi-supervised learning [18]. This kind of learning correspond to the union from the categories of supervised and unsupervised learning methods. In machine learning, these two techniques are fundamentally different. Unsupervised learning aims to seek inherent patterns in the unlabeled data set. The unsupervised learning techniques are directly related to density estimation problem in statistics, which aims to estimate the density function for a set of observed data.

Supervised learning aims to discover a *x* to *y* mapping given a training set containing pairs (*x*_*i*_,*y*_*i*_), where *y*_*i*_∈*Y* is called the label or *x*_*i*_ sample objective, and $Y = (y_{i})^{T}_{i \in [n]}$ represents the vector of labels in training data. Similar to the unsupervised learning, a requirement is that pairs (*x*_*i*_,*y*_*i*_) need to be collected independent and identically distributed [11].

The semi-supervised learning techniques make use of unlabeled data during training process. Generally, this learning could be used in contexts where there is a small amount of labeled data and a large amount of unlabeled data, such as the TIS prediction problem, in which the unlabeled data are the new molecules whose TIS has not been identified yet. Notice that, the TIS identification process usually requires the participation of a human expert or bio-chemical experiments, which makes the labeling process more expensive and complex. This reinforces the need for a technique that automates the identification of the TIS, as is the case of Transductive SVM (TSVM).

According with [17], the term “transductive” corresponds to a pattern recognition problem. It means that given the classifications *y*_*i*_,*i*=1,…,*l*, of *l* labeled samples *x*_*i*_,…,*x*_*l*_ from the training set, the goal is to discover the classification of the *k* unlabeled samples *x*_*l*+1_,…,*x*_*l*+*k*_ from the prediction set, contrary to the inductive inference, in which the goal is to find a function that can describe the problem and then classify the prediction set.

During the transductive learning training process the algorithm has access to the *l* training vectors *X*_*train*_, its labels *Y*_*train*_ (Eq. 1), and the *u* unlabeled prediction samples *X*_*test*_ (Eq. 2)

1 $$\begin{array}{*{20}l} X_{train} &= {x_{t_{1}},\ldots,x_{t_{l}}} \qquad Y_{train}={y_{t_{1}},\ldots,y_{t_{l}}} \end{array} $$

2 $$\begin{array}{*{20}l}  X_{pred}&={x_{p_{1}},\ldots,x_{p_{u}}}. \end{array} $$

The sets *X*_*train*_,*Y*_*train*_, and *X*_*pred*_ are used by the transductive learning in order to predict the labels of the prediction samples (Eq. 3).

3 $$ Y_{pred}^{*}={y_{p_{1}}^{*},\ldots,y_{p_{u}}^{*}},  $$

The goal is to minimize the ratio of incorrect predictions (Eq. 4) for the prediction.

4 $$ Err_{pred}(Y_{pred}^{*}) = \frac{1}{u} \sum_{i \in S_{pred}} \delta_{\frac{0}{1}} (Y_{i}^{*},Y_{i})  $$

where $\delta _{\frac {0}{1}}(Y^{*}_{i},Y_{i})$ is 0 if $Y^{*}_{i} = Y_{i}$ or 1 otherwise.

As previously mentioned, inductive methods are often used in the TIS prediction, differently from the transductive methods application that has not been discussed in the context. Note that the main purpose of the TIS prediction is to correctly identify positive AUG triplets (TIS) and not necessarily to identify an inductive function that represents the problem. It is important to enhance that, the use of inductive methods for new molecules may fail, since the new sequences may have different characteristics concerning the TIS prediction in comparison to the sequences used during the training process to obtain the model. On the other hand, transductive methods readjust the model for each new sequence to be predicted. Thus, it is relevant to consider and analyze the application of transductive inference to the TIS prediction problem.

Consequently, this work compares the behavior of the Transductive SVM (TSVM) and Inductive SVM (ISVM) applied to the TIS identification problem. For this, we consider two scenarios in relation to the training set. The first considers 90% of dataset for training and 10% for validation; and in the second scenario it was considered 10% for training and 90% for validation. The results show that the proposed approach based on transductive inference provides better results for organisms with smaller number of molecules (*Rattus norvegicus* and *Mus musculus*) in *F-measure* and *sensitivity* in comparison with the inductive method for predicting the TIS. The methods were tested with the molecules from the most representative organisms contained in the RefSeq database: *Rattus norvegicus*, *Mus musculus*, *Homo sapiens*, *Drosophila melanogaster* and *Arabidopsis thaliana*. The transductive method presented *F-measure* and *sensitivity* higher than 90% and also higher than the results obtained with ISVM.

This paper is organized as follows: Firstly, “Methods” section describes the databases considered in this study and the procedures used in the data preparation. The criterium for definition of the windows size for extraction of positive and negative sequences are analyzed and discussed. In this section the definition of the SVM parameters and the adopted validation process is presented. The “Results and discussion” section presents the results obtained by the comparative process between the ISVM and TSVM classifiers and a comparative study with the Netstart, TISHunter and TIS Miner programs. Finally, the “Conclusions” section presents the final considerations.

## Methods

This section presents the procedures carried out to evaluate the inductive and transductive inferences for TIS identification. For this, we describe the used databases to perform the tests, the window size definition, extraction process of positive and negative sequences, the definition of the SVM parameters and the evaluation metrics.

**Fig. 2:**
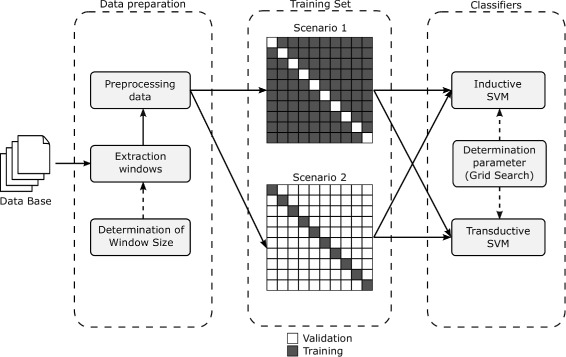
ISVM and TSVM evaluation methodology towards the solution for the TIS prediction problem schematically represented

Figure 2 schematically shows the methodology used in this work, illustrating all activities performed to investigate the TSVM behavior for the TIS prediction problem and to compare the ISVM and TSVM methods. This methodology will be described in the next sections.

### Materials

The used databases in our experiments (see Fig. 2) were extracted from the public repository RefSeq [14] from the NCBI (National Center for Biotechnology Information)^2^ on 22nd April 2014 referent to the following organisms: *Rattus norvegicus* (1383 molecules), *Mus musculus* (1097 molecules), *Homo sapiens* (21,528 molecules), *Drosophila melanogaster* (27,764 molecules), *Caenorhabditis elegans* (26,066 molecules) and *Arabidopsis thaliana* (35,173 molecules), which represents 96.07% of the molecules available in this repository. The remaining 3.93% molecules available in the RefSeq database (distributed among 14 organisms) were not considered in our study because it doesn’t generate a sufficient sequence for training the classifiers. For example, considering our methodology was possible to extract only 23 positive sequences and 18 negative sequences for the *Nasonia vitripennis* organism. Notice that this sequences number, in general, is not sufficient for a training process of classifiers.

Although the organism *Caenorhabditis elegans* have a large number of molecules, it could not be analyzed due to the fact that its molecules contain only the CDS region. In other words, this organism does not have a *upstream* region sufficient for our methodology.

Each molecule was identified according to the inspection level and classified as: *Model*, *Inferred*, *Predicted*, *Provisional*, *Reviewed*, *Validated* and WGS^3^. In this work we have considered only mRNA molecules with inspection level *Reviewed* since those records undergo a thorough review process.

### Window size definition

In this section the criteria to define the size of the analysis window will be discussed, which corresponds to the data preparation stage comprised by methodology proposed in this work (see Fig. 2).

According to the experiments carried out by [6, 11], the size of the nucleotide sequences extraction window directly influences the quality of the prediction model. A preliminary study, [6] indicates that asymmetric sized windows provide higher accuracy to the prediction model. Consequently, our work adopts asymmetric windows and the *upstream* region with the fewer amount nucleotides. This will be discussed bellow.

In order to define the amount of nucleotides in the *upstream* region, we have considered the ribosome scanning model and the Kozak consensus [3], which identifies a conservative pattern in the -6, -5, -4, -3, -2, -1, +1, +2, +3 and +4 positions (GCC**[A or G]**CCAUG**[G]**), where there is a predominance of nucleotides **[A or G]** and **[G]**, respectively, in the positions -3 and +4. A higher number of nucleotides in the *upstream* region was used by [1], in which -7 was identified as a conservative position. For the experiments carried out in our work, we use windows with 9 nucleotides in the *upstream* region, since the scanning model of the mRNA chain is made at each 3 nucleotides and guarantees that our analyses includes the previously identified conservative positions. In addition, our methodology avoids the unnecessary elimination of sequences when considering a small *upstream* region.

To define the amount of nucleotides in the *downstream* region, we have taken into account the results obtained by [1] and [13] where the authors suggest the existence of a pattern to define the TIS present in the CDS region of a molecule. In [13], the authors consider windows with size of 150 nucleotides in *downstream* region for the tests into database used by [12] and 270 nucleotides in *downstream* region for the validation in Human mRNAs. However, these sizes were empirically defined for the used databases and do not take into account the possibility of protein pattern in the *downstream* region.

**Fig. 3:**
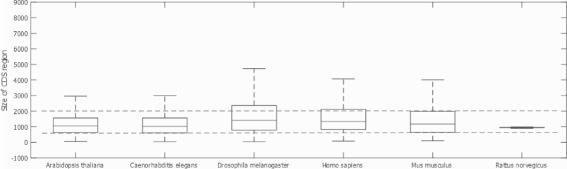
*Box* plot for the CDS region size per organism

Aiming to evaluate the existence of such pattern for the TIS in the *downstream* region, we have varied the amount of nucleotides in this region to be considered through an analysis of the CDS sizes from the studied organisms. Figure 3 depicts a box plot of the CDS sizes found in each organism. For the sake of readability, we have eliminated typical outliers from this type of graphic. CDS sizes in the range of values limited by the box represent 75% of all CDS sizes found in each organism. Therefore, the choice for the amount of nucleotides in the *downstream* region close to the CDS size may impact in classifier’s performance because most of the information from this region would be considered. Figure 3 shows that most of the evaluated molecules present CDS region with sizes ranging from 800 to 2000 nucleotides, limits shown as a dashed line. The *Drosophila melanogaster* organism has CDS region bigger than 2000, however windows with more than 2000 nucleotides prevent the study of organisms with fewer molecules, such as *Arabidopsis thaliana*.

To define a common amount of nucleotides in the *downstream* region to be applied for all studied organisms, we have identified in the Fig. 3 that the CDS region from the organism *Mus musculus* is mostly distributed from 800 to 2000 nucleotides. Defining the amount of nucleotides in the *downstream* region inside this interval enables to consider much of the information contained in the CDS region from the remaining organisms.

**Fig. 4:**
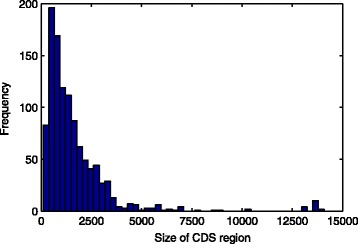
Frequency histogram of the intervals in the size of the CDS region from *Mus musculus*

In order to identify the amount of nucleotides in the *downstream* region, we have analyzed the frequency histogram of *Mus musculus* organism (see Fig. 4), which the intervals smaller than 2000 can be seen in the Table 1. The frequency histogram has been generated using package *fdth* ^4^ from R version 2.12.2.

**Table 1:** Frequency histogram of the intervals in the size of the CDS region from *Mus musculus*

Interval	[94,376)	[376,659)	[659,941)	[941,1220)	[1220,1510)	[1510,1790)	[1790,2070)
Relative frequency (%)	7.59	18.21	15.46	10.98	10.34	7.59	5.76
Median	235	518	800	1081	1365	1650	1930

We have defined the amount of nucleotides in the *downstream* region as the median from the interval of each class based on the frequency histogram of the size of the CDS region for *Mus musculus* (Table 1). We have eliminated the class with median 1930 because our preliminary experiments with this window size did not generate a representative size of training set for the organism *Rattus norvegicus*. Although the first two intervals are outside the range from 800 to 2000, these were considered in the analysis. Doing so, we evaluate the interference in the performance of the classifier when there is more information available regarding the CDS region. Therefore, 235, 518, 800, 1081, 1365 and 1650 are amount of nucleotides in the *downstream* region for the extraction window.

### Extraction of positive and negative sequences

For each window size previously established in the previous Section, the sequences were extracted using the developed program Transdutis^5^. A negative sequence (nTIS) can be differentiated according to its location, *upstream* or *downstream*, and with regards to the ribosome reading phase [3]. In this work we only consider windows in which the AUG is at most until the end of the CDS region. Therefore, we guarantee that all sequences used to generate the classification model have at least a portion of the CDS region, which supposedly contains a pattern to predict the TIS [13].

**Fig. 5:**
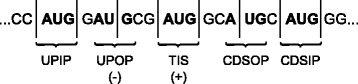
A sequence of an mRNA with the identification of the regions

The nTIS sequences locate in the *upstream* region in the reading phase of TIS [5] are classified as *upstream in phase* (UPIP) and those out of the reading phase of TIS are called *upstream out of phase* (UPOP). On the other hand, sequences locate in the CDS region in the reading phase of TIS are classified as CDS in phase (CDSIP) and those out of the reading phase of TIS are called CDS out of phase (CDSOP), as shown in Fig. 5.

Preliminary experiments using negative sequences (nTIS) UPIP, CDSOP, CDSIP as input to the SVM resulted in relatively low *F-measure* results, around 70% for the organism *Mus musculus*. Additionally, results from [13] indicate that UPIP sequences possess a very similar biological context to the TIS. These sequences may even start the protein translation process and be interrupted early on by the presence of a stop codon [8]. Thus, the sequences used as input for the inductive SVM (ISVM) and transductive SVM (TSVM) were only negative UPOP and positive TIS, as previously identified in Fig. 5.

During the sequence extraction process, we have preprocessed the database (see Fig. 2) in order to eliminate the duplicated sequences prioritizing the sequences from the (minority) positive class (TIS). The process of removing duplicated sequences consists in eliminating repeated occurrences of a sequence, thus the remaining sequences are named unique and the removed are name duplicated. Table 2 presents the amount of sequences extracted by window size, by organism and the number of duplicated sequences disregarded for training the classifier. Notice that, in general, the number of duplicated sequences found is greater for small window size and confirm the necessity of eliminating duplication.

**Table 2:** Amount of sequences extracted by classification and amount of duplicated sequences eliminated during the preprocessing

Downstream	TIS		UPSTREAM		UPSTREAM		CDS		CDS		
*Window*			out of Phase (nTIS)		in Phase		in Phase		out of Phase		
	Non-duplicated	Duplicated	Non-duplicated	Duplicated	Non-duplicated	Duplicated	Non-duplicated	Duplicated	Non-duplicated	Duplicated	
*Rattus norvegicus*											
235	113	49	123	61	58	34	11703	1373	9738	1989	
518	100	38	124	47	60	29	8630	1120	6161	1638	
800	81	29	114	41	58	28	2141	945	2170	1451	
1081	66	22	101	39	57	24	546	824	983	1278	
1365	48	14	86	37	54	15	463	741	822	1158	
1650	42	11	69	28	40	11	420	675	720	1056	
*Mus musculus*											
235	678	358	776	471	308	170	5154	4853	8364	8067	
518	581	272	810	384	315	147	4323	3779	7230	6316	
800	466	203	726	331	288	127	3612	2927	6102	5000	
1081	398	158	632	293	260	113	2976	2311	5318	3931	
1365	319	113	568	234	242	92	2506	1839	4440	3124	
1650	277	79	495	187	208	68	2104	1463	3757	2454	
*Homo sapiens*											
235	13564	7271	17729	9177	6972	3386	109658	109137	194726	192024	
518	13124	5674	18760	7188	7606	2492	94503	83527	171463	150192	
800	11579	4398	17917	5914	7334	1986	79368	63663	148148	117440	
1081	9716	3366	16085	4902	6629	1677	65717	48341	126260	90786	
1365	7753	2469	13662	3853	5649	1371	54818	37422	106842	71030	
1650	5877	1793	10918	2871	4537	1098	46233	29136	91066	56808	
*Drosophila melanogaster*											
235	15225	10455	26777	18065	12378	8252	142022	194250	200046	285816	
518	13723	9076	27548	16202	12787	7432	119185	162288	171884	244359	
800	11942	7745	26905	14748	12581	6704	99615	134106	146638	208787	
1081	10122	6594	25725	13314	12086	6092	82645	110225	124842	178443	
1365	8344	5400	23695	11929	11233	5474	69079	91113	106093	153047	
1650	6657	4390	21482	10472	10227	4740	58253	75705	90979	131754	
*Arabidopsis thaliana*											
235	20867	5157	15869	3515	6542	1319	196447	56135	388223	116238	
518	18440	9200	15663	2677	6555	975	145585	38519	299284	82624	
800	14948	3013	14112	2122	5968	750	105415	25929	221892	56195	
1081	11082	2046	11644	1592	4942	562	74236	17100	160512	38259	
1365	7683	1281	8453	1112	3658	399	51462	11625	115329	26194	
1650	4967	839	5952	808	2582	283	36505	8297	83812	18706	

Still regarding Table 2, CDS region contains higher number of duplicate sequences, which reinforces the possibility of existence of conservative information in this mRNA sequence region. Additionally, it is important to note a higher amount of nTIS sequences of type UPOP in comparison with UPIP sequences, indicating that these sequences are more representative, which justifies the choice made in this work.

In addition to equal sequences classified to the same class, there were also equal sequences differently classified, i.e., classified as TIS and nTIS in different molecules. This rarely occurs, mostly found in the organism *Drosophila melanogaster* in a proportion of about 1:5000 that corresponds to the total amount of extracted sequences. In this work, we disregarded those sequences differently classified.

TIS prediction problem is essentially unbalanced because for each analyzed molecule there exist only one TIS, with rare exceptions, of several AUG codons, whose do not start the protein translation. However, as presented in Table 3 (column TIS/nTIS), this problem has been alleviated by eliminating duplicates and using only out of phase negative *upstream* sequences (UPOP). Still, it is important to note that the amount of available TIS sequences is higher than the amount of nTIS sequences for windows of size 235, 518 and 800 nucleotides in the *downstream* region for the organism *Arabidopsis thaliana*.

**Table 3:** Amount of sequences after the elimination of duplicated sequences

Downstream	*Rattus norvegicus*			*Mus musculus*			*Homo sapiens*			*Drosophila melanogaster*			*Arabidopsis thaliana*		
*Window*	TIS	nTIS	TIS/nTIS	TIS	nTIS	TIS/nTIS	TIS	nTIS	TIS/nTIS	TIS	nTIS	TIS/nTIS	TIS	nTIS	TIS/nTIS
235	113	123	0.9187	678	776	0.8737	13564	17729	0.7651	15225	26777	0.5686	20867	15869	1.3150
518	100	120	0.8334	581	810	0.7173	13124	18760	0.6996	13723	27548	0.4981	18440	15663	1.1773
800	81	114	0.7105	466	726	0.6419	11579	17917	0.6462	11942	26905	0.4438	14948	14112	1.0592
1081	66	101	0.6535	398	632	0.6297	10122	25725	0.3935	10122	25725	0.3935	11082	11644	0.9517
1365	48	86	0.5581	319	568	0.5616	8344	23695	0.3521	8344	23695	0.3521	7683	8453	0.9089
1650	42	69	0.6087	277	495	0.4586	5877	10918	0.5383	6657	21482	0.3099	4967	5952	0.8345

Besides the duplicated sequences, we have eliminated sequences containing windows longer than amount of nucleotides existent in the molecule for both *upstream* and *downstream*.

Similar to [4, 19], the sequences were codified as binary chain, i.e., 4 bits to represent each nucleotide A, C, G and U as 1000, 0100, 0010 and 0001, respectively.

### SVM parameter definition

Another stage of the proposed methodology is to define the parameters of the SVM algorithms to be used in the ISVM and TSVM classifiers. This activity is directly linked to the training process, as can be seen in Fig. 2.

For the non-linearly separable problems, as in the TIS prediction, it is necessary to use variables that smoothen the optimization problem restrictions, allowing the occurrence of some misclassification and the use of a kernel function in order to map the training data to specific space. Parameter C, known as penalty parameter, determines the weight attributed to each incorrect classification provided by the classifier, so that the higher the value the more specific classifier and more intolerant to incorrect classification.

The efficiency of those two classifiers depends on the proper selection of the parameters of the kernel function and the optimal hyperplane separation margin’s smoothing parameter, represented by *C*. Our work uses the Gaussian RBF (Radial Basis Function) kernel function (Eq. 5) and its parameters defined as *σ*, that corresponds to the variation of Gaussian function. However, our work uses the parameter *γ* as commonly found in implementations of SVM classifiers, which is defined as $\gamma =-\frac {1}{2\sigma ^{2}}$.

5 $$ K(x_{i},x_{j}) = \exp^{\gamma\parallel x_{i} - x_{j} \parallel^{2}}  $$

The parameters were defined using the *Grid search* method [20] implemented in the *libsvm* ^6^. This method defines a optimal set of parameters by an exhaustive search within a predefined range of values for each parameter. Preliminary experiments with this method using all the 1454 sequences from *Mus musculus* for a window size of 235 (see Table 3). It was required about 5 hours of processing in order to find the best pair of parameters (*C*,*γ*). The experiment was executed in a high-performance SGI Altix server in the National Supercomputing Center at Federal University of Rio Grande do Sul^7^.

Due to the high amount of available molecules (around 20 thousand) for the remaining analyzed organisms and the *Grid Search’s* high runtime (given by the SVM’s execution time and the amount of records in the training set), we use 10% of the available sequences. Those sequences were chosen using the *Mersenne Twister* method [21], but keeping the ratio of positive (TIS) and negative (nTIS) classes. *Grid Search* was executed for each of the organisms and window size defined in Table 3. See the Additional file 1 for the values for the parameters (*C*,*γ*) found by the *Grid Search* using RBF kernel function, which were used for the training of ISVM and TSVM.

The assessment of the results was performed using $Precision = 100 \times \frac {TP}{TP+FP}$, $sensitivity = 100 \times \frac {TP}{TP+FN}$, $F-measure = 2 \times \frac {Precision \times sensitivity}{Precision + sensitivity}$ metrics (where TP = True Positive, TN = True Negative, FP = False Positive and FN = False Negative) and ROC (Receiver Operating Characteristic Curve) [22].

### Validation process

We have applied the 10-fold cross-validation method, which guarantees the statistical validation of the model. It consists of subdividing the available data set in 10 folds of the same size from which 9 are used for training the remaining one for validation.

However, this validation process induces a favorable context to the inductive learning techniques because 90% (9 folds) of the available data goes for training and the remaining one (10%) for the validation. Thus, in order to compare the performance of ISVM and TSVM in a more balanced context, we have proposed experiments in two different scenarios.

From now on the traditional cross-validation will be referenced as Scenario 1. The usage of the Scenario 1 is valid in order to evaluate the transductive classifier in an unfavorable context. However, it is important to evaluate which the best context is to apply each of the inferences. Consequently, we propose a variation of the cross-validation method to simulate a context in which the available data for training are scarce. It aims to invert the cross-validation model, e.g., 10% (1 fold) of the data are available for the training and the remaining 90% for the model validation. From now one this scenario is called Scenario 2. Data from both Scenario 1 and 2 are used for training the ISVM and TSVM (refer to Fig. 2).

## Results and discussion

This experiments aims to analyze the behavior of ISVM and TSVM for the TIS prediction problem. As previously described this analysis was performed using 6 window sizes for sequence extraction in two different scenarios, in which the amount of available sequences is different.

Table 4 presents the *precision* obtained for both methods, ISVM and TSVM. It is possible to observe that the *precision* of the ISVM and TSVM is similar for both scenarios, with few exceptions. The largest differences are found in the *Rattus norvegicus* and *Mus musculus* organisms, which have few training sequences (see Table 3).

**Table 4:** Validation *precision* results using ISVM and TSVM methods for the Scenarios 1 and 2

Downstream	Scenario 1		Scenario 2	
*Window*	ISVM (inductive)	TSVM(transdutivo)	ISVM (inductive)	TSVM (transdutivo)
*Rattus norvegicus*				
235	79.22±2.71	81.13±5.08	69.81±4.45	72.00±2.72
518	89.00±5.51	89.33±3.03	82.33±3.05	79.52±2.72
800	90.69±4.23	89.07±3.65	94.37±3.01	84.71±2.43
1081	89.40±8.60	89.66±5.26	97.66±4.33	79.48±3.99
1365	96.00±4.95	85.00±7.59	88.00±3.71	77.58±3.45
1650	100.00±0.0	93.57±6.32	100.00±0.0	77.31±3.42
*Mus musculus*				
235	88.51±2.36	87.29±0.93	83.32±1.04	84.32±0.55
518	93.41±1.45	93.37±1.46	93.17±1.10	92.45±0.66
800	98.23±0.80	97.38±0.80	97.86±0.48	93.68±0.47
1081	99.20±0.75	97.94±1.18	98.68±0.42	95.45±0.55
1365	99.35±1.19	98.70±0.97	99.57±0.20	96.16±1.20
1650	99.62±0.68	99.25±0.91	99.69±0.19	96.84±1.55
*Homo sapiens*				
235	91.99±0.43	90.48±0.16	90.50±0.30	87.41±0.11
518	96.15±0.24	94.98±0.11	95.72±0,09	94.97±0.54
800	97.83±0.17	97.69±0.06	97.55±0.24	96.57±0.05
1081	98.03±0.33	97.69±0.11	97.85±0.23	97.42±0.04
1365	98.81±0.23	98.43±0.10	98.52±0.21	98.08±0.06
1650	99.04±0.31	98.76±0.13	98.63±0.22	98.39±0.06
*Drosophila melanogaster*				
235	93.38±0.38	93.46±0.20	91.97±0.35	90.32±0.07
518	95.74±0.34	95.75±0.13	95.37±0.17	94.47±0.06
800	96.73±0.28	96.92±0.06	96.57±0.30	95.53±0.06
1081	96.86±0.26	96.74±0.07	96.76±0.25	96.20±0.08
1365	97.23±0.41	97.07±0.14	97.33±0.17	96.65±0.08
1650	97.71±0.27	97.93±0.12	97.64±0.27	96.57±0.16
*Arabidopsis thaliana*				
235	93.10±0.22	93.73±0.26	91.39±0.16	92.77±0.06
518	97.05±0.28	97.50±0.13	96.30±0.10	97.26±0.04
800	98.16±0.20	98.58±0.13	97.84±0.05	98.46±0.04
1081	98.76±0.20	98.96±0.09	98.50±0.04	99.06±0.02
1365	99.03±0.17	99.31±0.14	98.85±0.09	99.32±0.02
1650	99.22±0.02	99.54±0.14	99.18±0.05	99.35±0.07

For the Scenario 2, in which only 10% of the sequences are available, the *precision* of both classifiers is smaller, as expected. It is important to observe that the greater the number of training sequences for an organism the greater the *precision* obtained with ISVM and TSVM classifiers. However, for the Scenario 2, the *sensitivity* shown in the Table 5 indicates that the ISVM classifier falls by identifying the TIS. This occurs for *Rattus norvegicus* and *Mus musculus* organisms, which have few molecules.

**Table 5:** Validation *sensitivity* results using ISVM and TSVM methods for the Scenarios 1 and 2

Downstream	Scenario 1		Scenario 2	
*Window*	ISVM (inductive)	TSVM(transdutivo)	ISVM (inductive)	TSVM (transdutivo)
*Rattus norvegicus*				
235	81.30±8.20	78.77±4.36	61.63±4.69	72.00±2.73
518	88.00±8.67	91.00±3.33	59.89±6.90	79.67±2.73
800	88.89±4.16	88.89±4.16	34.66±10.69	84.63±2.38
1081	82.50±13.15	85.83±6.55	18.01±14.06	78.42±4.01
1365	79.17±13.71	82.5±7.10	11.41±13.73	75.03±3.37
1650	81.66±10.27	95.00±6.19	7.93±2.63	77.44±3.77
*Mus musculus*				
235	83.88±3.94	87.16±0.84	76.93±1.51	84.21±0.45
518	90.97±1.58	92.77±1.50	81.75±1.45	92.36±0.39
800	95.28±2.43	96.97±0.66	78.16±2.35	93.72±0.49
1081	95.70±1.74	97.94±1.18	79.54±1.47	95.53±0.59
1365	96.58±1.58	97.94±1.49	67.05±3.67	96.16±0.78
1650	97.40±2.06	98.35±1.78	65.25±3.94	96.74±0.65
*Homo sapiens*				
235	88.72±0.44	82.83±0.33	90.52±0.28	87.42±0.11
518	95.26±0.25	91.92±0.26	95.71±0.08	94.69±0.17
800	97.17±0.20	94.12±0.18	97.53±0.26	96.57±0.08
1081	97.74±0.27	95.89±0.19	97.84±0.23	97.44±0.04
1365	98.31±0.30	96.47±0.13	98.52±0.21	98.09±0.04
1650	98.33±0.35	96.61±0.22	98.60±0.24	98.41±0.07
*Drosophila melanogaster*				
235	90.28±0.46	85.98±0.30	91.96±0.34	90.33±0.07
518	94.98±0.23	91.98±0.25	95.38±0.17	94.48±0.06
800	96.38±0.16	93.01±0.17	96.57±0.30	95.54±0.06
1081	96.80±0.38	94.82±0.21	96.76±0.25	96.21±0.08
1365	97.36±0.45	95.38±0.23	97.31±0.18	96.66±0.07
1650	97.32±0.39	94.42±0.28	97.70±0.30	96.57±0.16
*Arabidopsis thaliana*				
235	94.74±0.37	93.75±0.27	94.10±0.14	92.76±0.05
518	98.13±0.17	97.50±0.13	97.73±0.09	97.26±0.04
800	99.25±0.10	98.57±0.12	99.01±0.05	98.46±0.04
1081	99.38±0.10	98.94±0.10	99.24±0.06	99.06±0.02
1365	99.48±0.13	99.30±0.14	99.44±0.08	99.32±0.03
1650	99.68±0.18	99.48±0.21	99.44±0.11	99.35±0.08

With the evaluation of *precision* and *sensitivity* separately, we just have a partial idea of which classifier is better for the prediction of TIS problem. So, the *F-measure* metric (the harmonic mean of *sensitivity* and *precision*) was used to compare the performance of the classifiers (ISVM and TSVM) taking into account both *precision* and *sensitivity*. Table 6 presents the *F-measure* results that point the TSVM is better than ISVM for the organisms that have fewer molecules, in this case the organism *Rattus norvegicus* and *Mus musculus*. This results reinforce that TSVM is more indicated for organisms that have fewer molecules or are under studied.

**Table 6:** Validation *F-measure* results using ISVM and TSVM methods for the Scenarios 1 and 2

Downstream	Scenario 1		Scenario 2	
*Window*	ISVM (inductive)	TSVM(transdutivo)	ISVM (inductive)	TSVM (transdutivo)
*Rattus norvegicus*				
235	79.88±5.07	79.89±4.58	65.04±3.19	71.99±2.72
518	88.00±6.42	90.09±2.77	69.00±4.78	79.53±2.33
800	89.44±2.39	88.92±3.71	46.96±12.52	84.64±2.16
1081	84.58±10.31	87.51±5.46	25.05±12.10	84.64±2.87
1365	84.06±9.84	83.57±6.94	14.84±13.31	76.00±1.91
1650	88.95±6.52	94.23±6.12	14.43±4.36	77.15±2.57
*Mus musculus*				
235	86.04±2.80	87.23±0.88	79.96±0.81	84.26±0.50
518	92.13±0.84	93.06±1.42	87.05±0.69	92.40±0.38
800	96.68±1.26	97.17±0.69	86.85±1.40	93.70±0.41
1081	97.40±1.09	97.94±1.18	88.06±0.90	95.49±0.57
1365	97.93±1.17	98.30±1.05	79.99±2.56	96.14±0.52
1650	98.47±1.17	98.78±1.16	78.70±2.81	96.76±0.68
*Homo sapiens*				
235	90.32±0.29	86.49±0.13	90.51±0.29	87.42±0.11
518	95.71±0.11	93.43±0.13	95.72±0.08	94.83±0.32
800	97.50±0.15	95.87±0.08	97.54±0.25	96.57±0.06
1081	97.89±0.20	96.78±0.09	97.85±0.23	97.43±0.04
1365	98.56±0.18	97.44±0.07	98.52±0.21	98.09±0.05
1650	98.69±0.23	97.68±0.11	98.62±0.23	98.40±0.06
*Drosophila melanogaster*				
235	91.81±0.38	89.56±0.09	91.97±0.34	90.33±0.07
518	95.36±0.21	93.82±0.07	95,38±0.17	94.48±0.06
800	96.56±0.17	94.93±0.07	96.57±0.30	95.54±0.06
1081	96.83±0.24	95.77±0.08	96.76±0.25	96.21±0.08
1365	97.30±0.31	96.22±0.09	97.32±0.17	96.66±0.07
1650	97.52±0.16	96.14±0.13	97.67±0.28	96.57±0.14
*Arabidopsis thaliana*				
235	93.91±0.18	93.73±0.27	92.73±0.04	92.76±0,05
518	97.59±0.16	97.50±0.13	97.01±0.05	97.25±0.04
800	98.70±0,11	98.57±0.13	98.42±0.02	98.45±0.04
1081	99.06±0.14	98.95±0.09	98.86±0.03	99.05±0.02
1365	99.25±0.10	99.30±0.14	99.14±0.04	99.31±0.02
1650	99.44±0.16	99.50±0.17	99.30±0.05	99.35±0.04

**Fig. 6:**
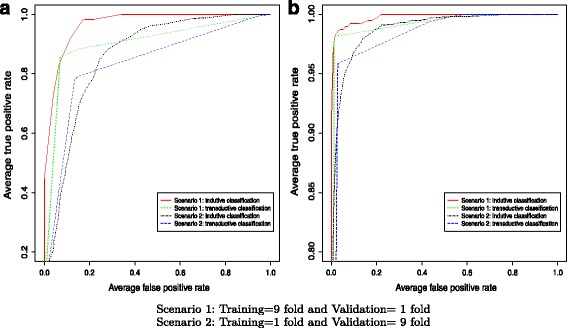
ROC *curve* for **a** *Rattus norvegicus* and **b** *Mus musculus* organisms

We further evaluated the performance of ISVM and TSVM classifiers by ROC curves. Figure 6 a and b illustrate the ROC curves for *Rattus novergicus* and *Mus musculus* organisms, respectively. As already discussed, in Scenario 2, the TSVM classifier is better than the ISVM classifier (Fig. 6 a). Although the area under the ROC curve, in Scenario 2, is slightly smaller for the transductive classifier (AUC = 0.837 in the transductive and 0.917 in the inductive for *Rattus norvegicus* organism), the best classification model, the one that is closest to the point (0,100%), that is, with a higher true positive rate and lower false positive rate, is obtained by TSVM classifier.

On the other hand, when considering the inductive scenario (Scenario 1), with a higher number of training sequences, the inductive classifier presented better results than the transductive one. This conclusion is based on the area under an ROC curve, AUC = 0.973 in the inductive and 0.917 in the transductive for *Rattus norvegicus* organism. The same behavior was observed for *Mus musculus* organism (Fig. 6 b).

Another important results refers to the size of the analyzed extraction windows. By analyzing the *F-measure* results (see Table 6) it is possible to notice that the greater the number of nucleotides in the *downstream* region of the extraction window the better the performance of the classifiers. Nevertheless, there is a similar performance for windows with 1081, 1365 or 1650 nucleotides in the *downstream* region. On the other hand, there is a considerable reduction in amount of available sequences for training (see Table 3). Therefore, for the evaluated organisms, it is appropriate to use as window size the smallest among the largest. In this work, we consider 1081 nucleotides in the *downstream* region, regardless the organism.

By analyzing these results it is possible to observe that the usage of the TSVM method better suits organisms with few labeled sequences, e.g., *Rattus norvegicus* and *Mus musculus* organisms. When using ISVM comes a question, for how long the inductive classifier is valid? To handle with this situation, it is necessary retraining the classifier constantly in order to ensure its accuracy and representativeness, since the frequency in which new sequences (intrinsically different from the sequences considered in the original training set) are included in the database may compromise the classifier’s performance.

Although the TSVM classifier, by the transductive principle itself, needs to be readjusted for each new sequence, there is an increase in the reliability of the classification process. This readjust is justified when the organisms have few sequenced molecules. The retraining implies an increase in the computational cost in comparison to inductive methods. However, this cost can be reduced if each readjustment process considers the SVs of the previous readjustment in addition the new sequences.

Table 7 presents the amount of SV used in the TSVM approach and the elapsed time for the classification of one molecule from each organism.

**Table 7:** TSVM’s retraining computational cost

Organism	Amount of SV	Time (s)
*Rattus norvegicus*	165	2
*Mus musculus*	544	6
*Homo sapiens*	4275	759
*Drosophila melanogaster*	4537	1175
*Arabdopsis thaliana*	3188	219

### Comparative study

In order to compare our approach in a real scenario of TIS identification, the next stage of this work is to perform a comparative analysis among some of the main programs for TIS prediction.

For comparative study, a test sets, which was not included in the training of the ISVM and TSVM classifiers, were utilized. This new database comprises data from RefSeq extracted between 22 April and 22 September, 2014.

The test sets have the following number of molecules for each considered organism: *Rattus norvegicus* (125 molecules), *Mus musculus* (36 molecules), *Homo sapiens* (113 molecules), *Drosophila melanogaster* (106 molecules) and *Arabidopsis thaliana* (15 molecules).

The considered programs in this evaluation are the following: TISHunter [13], TIS Miner [11], NetStart [12] and TransduTIS, developed for this work, which implements the inductive (TransduTIS-I) and transductive (TransduTIS-T) approaches.

We developed a python script^8^ to automate the tests with TisHunter, TIS Miner and NetStart. To evaluate TISHunter, we have used the URL^9^ to submit each mRNA for testing with the default settings. The TIS Miner program was evaluated using the URL^10^ with default parameters, with the number of predictions set to maximum value. We used a classification threshold of 0.6 for this program, such that for each AUG with score greater then 0.6 we consider a positive prediction; otherwise, if score is fewer then 0.6 we consider a negative prediction. Finally, to evaluate the NetStart we used the URL^11^ and setting its parameters to vertebrate. All the tests are available at ^8^.

Both ISVM and TSVM were tested with extraction windows of 1090 nucleotides (1081 in the *downstream* region and 9 in the *upstream* region). Molecules that did not meet these conditions were not considered in the tests.

Table 8 presents the results of the tests for each studied organism. We also present the amount of hit and not hit for each tool analyzed. Hit corresponds to AUG that is TIS and was classified as TIS, and not hit corresponds to AUG that is TIS but was classified as nTIS. It is important highlight that TISHunter is essentially predictor, so it was not possible to infer information about the classification process to build a confusion matrix. For calculation of the hit and not hit, only occurrences of AUG in the *upstream* region were considered.

**Table 8:** Comparison among methods

	*Rattus norvegicus*		*Mus musculus*		*Homo sapiens*		*Drosophila melanogaster*		*Arabidopsis thaliana*	
Method	Hit	not Hit	Hit	not Hit	Hit	not Hit	Hit	not Hit	Hit	not Hit
TransduTIS-I	109	16	22	14	102	11	95	11	15	0
TransduTIS-T	122	3	36	0	107	6	105	1	15	0
TISHunter	112	13	35	1	106	7	93	13	14	1
TIS Miner	89	36	34	2	91	22	76	30	12	3
NetStart	109	16	31	5	84	29	78	28	5	10

TransduTIS-I and TransduTIS-T are, respectively, the inductive and transductive approaches developed in this work

By analyzing the results, we have observed that the TransduTIS-T has the best hit and not hit among the evaluated tools. It means that the herein proposed model was able to better characterize the context of TIS prediction, which is important aiming to identify the higher possible amount of AUG codons that are truly TIS. Thus, researchers in TIS identification may more safely analyze proteins generated from this identification. The TISHunter program [13], which uses Edit Kernel functions, obtained significant results as well, reinforcing the hypothesis of conservative features in the CDS region to the TIS prediction.

## Conclusions

In this paper we compare the Inductive (ISVM) and Transductive (TSVM) classification methods for TIS identification. We describe the sequence extraction process, the preprocessing adopted and the elimination of duplicate sequences, which are important aspects for TIS prediction. We also present an approach to not incur the unbalancing, common situation in TIS identification. Besides, we have demonstrated the viability by using asymmetric extraction windows with a large amount of nucleotides in the *downstream* region.

The results show that the TSVM approach ensured an improvement, specially in *F-measure* and *sensitivity*, for organisms that have a small amount of mRNA molecules, as observed in the *Rattus norvegicus* and *Mus musculus* organisms. For organisms with a larger number of sequences, the inductive approach is recommended. When compared with other tools, in a real scenario of TIS identification, the transductive approach proved to be efficient for TIS identification in mRNA molecules.

Although the proposed methodology has achieved satisfactory results, some limitations can be mentioned: first, the sequences extraction process depends of a window fixed size, in both the *upstream* and *downstream* regions. This limits the classification of some molecules, as observed in *Caenorhabditis elegans* organism, which has a small *upstream* window. Another observed aspect corresponds to retraining process of the TSVM classifier, when it is desired to identify the TIS of new molecules.

Finally, this work provides a web interface, TransduTIS-I and TransduTIS-T, for the identification of TIS.

## Endnotes

^1^ Available at http://tishunter.ucr.edu/

^2^ Available at http://www.ncbi.nlm.nih.gov/

^3^ A description of each status is available at http://www.ncbi.nlm.nih.gov/books/NBK21091/

^4^ https://cran.r-project.org/web/packages/fdth/

^5^ Available at http://transdutis.com.br/

^6^ Available at https://www.csie.ntu.edu.tw/~cjlin/libsvm/

^7^ More information available at http://www.cesup.ufrgs.br

^8^ http://www.icei.pucminas.br/projetos/dsrgroup/?wpdmpro=transdutis

^9^ http://tishunter.ucr.edu/cgi-bin/tishunter.cgi

^10^ http://dnafsminer.bic.nus.edu.sg/cgi-bin/tis.pl

^11^ http://www.cbs.dtu.dk/cgi-bin/webface2.fcgi

## Additional file

Additional file 1 SVM parameters obtained by executing the Grid Search method Due to the high amount of available molecules (around 20 thousand) for the remaining analyzed organisms and the *Grid Search’s* high runtime (given by the SVM’s execution time and the amount of records in the training set), we use 10% of the available sequences. Those sequences were chosen using the *Mersenne Twister* method, but keeping the ratio of positive (TIS) and negative (nTIS) classes. *Grid Search* was executed for each of the organisms and window size defined in this work. This table presents the values for the parameters (*C*,*γ*) found by the *Grid Search* using RBF kernel function, which were used for the training of ISVM and TSVM. (XLS 28.0 kb)

## Abbreviations

CDS: Coding sequence
CDSIP: CDS in phase
CDSOP: CDS out of phase FN: False negatives
ISVM: Inductive SVM
mRNA: Messenger RNA
NCBI: National center for biotechnology information
nTIS: non-TIS
RBF: Radial basis function
RefSeq: Reference sequence
RNA: Ribonucleic acid
ROC: Receiver operating characteristic
SV: Support vectors
SVM: Support vector machine
TIS: Translation initiation site
TSVM: Transductive SVM
UPIP: UPstream in phase
UPOP: Upstream out of phase
URL: Uniform resource locator
